# Addressing huge spatial heterogeneity induced by virus infections in lentil breeding trials

**DOI:** 10.1186/s40709-016-0039-6

**Published:** 2016-03-01

**Authors:** Anastasia Kargiotidou, Dimitrios N. Vlachostergios, Constantinos Tzantarmas, Ioannis Mylonas, Chrysanthi Foti, George Menexes, Alexios Polidoros, Ioannis S. Tokatlidis

**Affiliations:** Department of Agricultural Development, Democritus University of Thrace, 68200 Orestiada, Greece; Industrial and Fodder Crops Institute, Hellenic Agricultural Organization, 41335 Larissa, Greece; School of Agriculture, Aristotle University of Thessaloniki, 54124 Thessaloniki, Greece

**Keywords:** Honeycomb method, Randomized complete block, Selection effectiveness

## Abstract

**Background:**

Spatial heterogeneity can have serious effects on the precision of field experimentation in plant breeding. In the present study the capacity of the honeycomb design (HD) to sample huge spatial heterogeneity was appraised. For this purpose, four trials were conducted each comprising a lentil landrace being screened for response to viruses.

**Results:**

Huge spatial heterogeneity was reflected by the abnormally high values for coefficient of variation (CV) of single-plant yields, ranging 123–162 %. At a given field area, increasing the number of simulated entries was followed by declined effectiveness of the method, on account of the larger circular block implying greater intra-block heterogeneity; a hyperbolic increasing pattern of the top to bottom entry mean gap (TBG) indicated that a number of more than 100 replicates (number of plants per entry) is the crucial threshold to avoid significant deterioration of the sampling degree. Nevertheless, the honeycomb model kept dealing with variation better than the randomized complete block (RCB) pattern, thanks to the circular shape and standardized type of block that ensure the less possible extra heterogeneity with expanding the area of the block.

**Conclusions:**

Owing to the even and systematic entry allocation, breeders do not need to be concerned with the extra spatial heterogeneity that might induce the extra surface needed to expand the size of the block when many entries are considered. Instead, they could improve accuracy of comparisons with increasing the number of replicates (circular blocks) despite the concomitant greater overall spatial heterogeneity.

## Background

Total phenotypic variation, the outcome of plant-to-plant phenotypic differences, comprises two main parts of variability, the genetic part and the acquired one. Soil heterogeneity is the main source of acquired variability inflated by uneven seed emergence, effects of clods and capping in wet soils, uneven application of applied inputs, differential effects of biotic and abiotic stresses, and interactions among these factors [1]. Spatial heterogeneity, reflecting the acquired part of the plant-to-plant variability, is a major concern in conventional breeding associated with low heritability and obstructing recognition of the outstanding genotypes [2–5]. Nevertheless, spatial heterogeneity cannot be eliminated entirely, being thus a ubiquitous feature of breeding trials. Avoidance of heterogeneous soils as well as measures to limit other operative events (e.g. even application of applied inputs) constitute a common practice. Further, different experimental configurations have been invented to tackle the problem, with the classical randomized complete block (RCB) being the most popular among breeders [6, 7]. In addition, application of modified experimental designs combined with suitable analysis models have been suggested with promising results under certain premises [8, 9].

Even though oxymoron, in case of breeding for tolerance to any biotic or abiotic stress the spatial heterogeneity induced by that stress might be desirable. Such kind of acquired variability might allow recognition of the susceptible genotypes and selection of the potentially tolerant ones. It has been reported that spaced plants intensify phenotypic expression of susceptibility with regard to biotic stresses in general [10] or insect-transmitted viruses in particular [11, 12]. In a lentil landrace, ultra-low density resulted in huge spatial heterogeneity due to insect-transmitted and seed-borne viruses [13, 14]. Under such adverse circumstances the pattern of experimental field configuration to perform progeny testing of the selected individuals is of paramount importance to sample the extraordinary spatial heterogeneity and accomplish precise evaluation.

From breeding perspective, growing individual plants widely apart to preclude any plant-to-plant interference for resources and ensure nil-competition has been asserted fundamental condition, leading to invention of the honeycomb breeding designs (HD) [15–18]. Nil-competition maximizes phenotypic expression and differentiation to facilitate genotype screening; it also copes with the suspending role of competition in the evaluation and selection of individual genotypes [1, 17]. Another main principle that distinguishes the honeycomb breeding model from the common conventional breeding schemes is that the individual plant rather than the classical plot (PL) is considered as experimental unit [18]. In a recent work, the honeycomb field experimental arrangement was found more efficient compared to the classical models of RCB, nearest neighbor adjustment and the lattice design to counteract the confounding effects of acquired variability on the single-plant performance of maize and wheat trials [19]. It was documented that essential principles of blocking, replication, and nearest-neighbor adjustment on the same baseline are its main characteristics. In honeycomb breeding, breeders do not need to be concerned with the pattern and orientation of soil variability in order to decide on the layout of the field plan, the shape, size and orientation of the PLs, and grouping of the PLs into blocks. Standardized configuration on account of even and systematic instead of random entry allocation makes the honeycomb model unique in sampling the acquired variability [13, 18, 19]. The objective of this study was to appraise the systematic entry allocation of the honeycomb configuration as a tool to effectively sample huge acquired variability mainly induced by virus infection. Intended situation of huge spatial heterogeneity for specific breeding purpose raises extra concern about objectiveness of entries’ comparative evaluation.

## Results

**Fig. 1:**
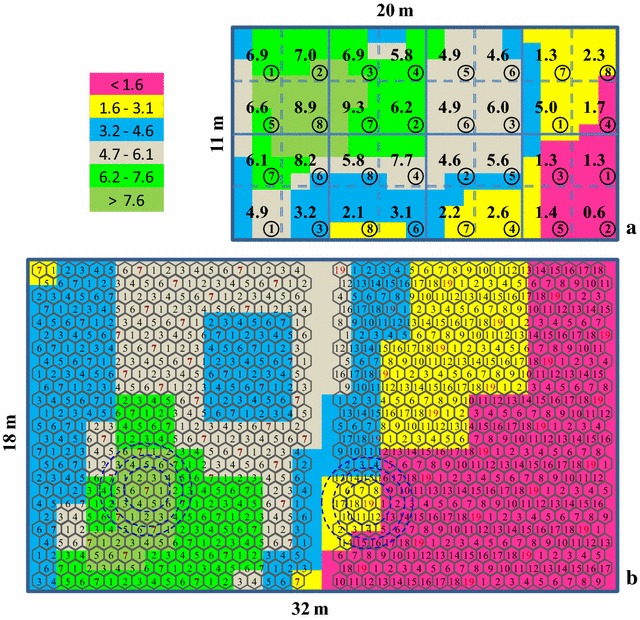
Smoothed uniformity map of the landrace ‘Lefkada’ trial at inter-plant distance of 0.50 m (**a**) and 0.80 m (**b**). The colored legend (*above left*) gives the corrected yield range of small plot units corresponding to the respective delineated area. At 0.50 m the whole area is divided into *32 square plots* including their actual mean yield per plant (g); the within-circle numbers represent the best scenario of randomization of eight entries into four complete blocks. At 0.80 m the potential honeycomb arrangement of seven entries (HD7) constructed on k value of 2 (*left*), as well as of 19 entries (HD19) constructed on k value of 7 (*right*) are illustrated (fully lengthwise and partly breadthwise). Entries are always positioned on a net pattern of *equilateral triangles*. Each *circle* of both sets corresponds to a fixed complete block of seven (*the interior*) or 19 (*the middle*) or 31 (*the exterior*) entries, evenly repeatable across the entire experimental area (Based on Fasoulas & Fasoula [18])

**Fig. 2:**
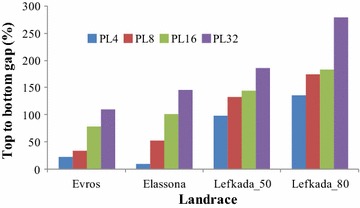
Top to bottom gap when each trial including a landrace split into four (PL4), eight (PL8), 16 (PL16) and 32 (PL32) plots

Information in terms of the two sets of trials analysed in this study is given in Table 1. Data quoted in Table 2 show that, as regards grain yield per plant landraces, ‘Evros’ and ‘Elassona’ averaged the same (3.35 g), while the first exhibited higher standard deviation (4.31 vs 4.10 g) and coefficient of variation (CV) (129 vs 123 %). Landrace ‘Lefkada’ yielded 30 % higher and resulted in 14 % lower CV at 0.50 compared to 0.80 m interplant distance (4.71 vs 3.61 g, and 140 vs 162 %, respectively). Spatial heterogeneity of ‘Lefkada’ is depicted in the uniformity map, with range of actual mean yields of 32 PLs being 0.6–9.3 g at 0.50 m (Fig. 1a) and 0.02–10.10 g at 80 cm (not shown). The top to bottom gap (TBG) consistently increased with increasing the number of PLs from 4 to 32, ranging from 22–110, 9–146, 98–186 and 136–279 % for ‘Evros’, ‘Elassona’, ‘Lefkada_50′ and ‘Lefkada_80’, respectively (Fig. 2).

**Table 1:** The landrace tested, year and location, soil properties, and details of the two sets of trials of the study

Landrace	Year/Location	Soil properties	Trial details^†^
	1st set		
Evros	2006–07/41^o^29′Ν, 26^ο^32′Ε, 25 m a.s.l. (Orestiada)	Silty clay with pH 7.6, organic matter 21.5 g kg^-1^, N-NO_3_ 10.1 mg kg^-1^, P-Olsen 13.5 mg kg^-1^ and K 171 mg kg^-1^	30 × 32 m/100 cm/34 rows × 32 plants/1088–584 plants
Elassona	As above	As above	30 × 32 m/100 cm/34 rows × 32 plants/1088–622 plants
	2nd set		
Lefkada	2011–12/39^o^36′Ν, 22^ο^25′Ε, 74 m a.s.l. (Larissa)	Clay loam with pH 7.8, organic matter 12.7 g kg^-1^, N-NO_3_ 39 mg kg^-1^, P-Olsen 10 mg kg^-1^ and K 150 mg kg^-1^	18 × 32 m/80 cm/25 rows × 40 plants/1000–848 plants
Lefkada	As above	As above	11 × 20 m/50 cm/25 rows × 40 plants/1000–894 plants

*a.s.l.* above sea level

^†^ Dimensions of the experimental area (length × width)/interplant distance/number of rows × number of single plant hills per row/established–harvested plants

**Table 2:** Number of harvested plants (n), mean yield per plant, the respective standard deviation (s) and coefficient of variation (CV)

Landrace	n	Yield per plant (g)	s (g)	CV (%)
Evros	584	3.35	4.31	129
Elassona	622	3.35	4.10	123
Lefkada-80	848	3.61	5.84	162
Lefkada-50	894	4.71	6.61	140

**Fig. 3:**
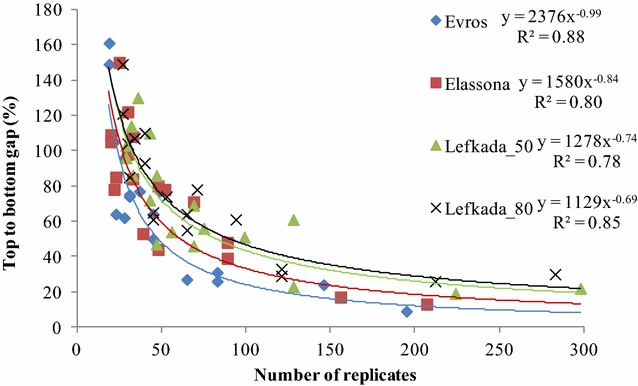
As the number of replicates (number of plants per entry) falls below 100 the top to bottom gap increases drastically and at statistically significant level (Table 3), illustrating that inclusion of at least 100 plants per entry is essential to sample the extreme spatial heterogeneity induced by virus infections in the honeycomb breeding trials of the lentil landraces ‘Evros’, ‘Elassona’, and ‘Lefkada’

In honeycomb analysis, increasing the number of simulated entries reflecting smaller entry size was accompanied by increased values of TBG (Table 3). Ranges of TBG were 9–161 % for ‘Evros’, 13–109 % for ‘Elassona’ and 14–94 % for their pooled data. For landrace ‘Lefkada’ the TBG ranges were 19–130 % at 0.50 m, 26–149 % at 0.80 m and 13–86 % with trials united. The entry size and the magnitude of TBG consistently exhibited a negative simple correlation coefficient (*r* = −0.72 up to −0.84), significant at *p* < 0.001. Their relationship is illustrated in Fig. 3 indicating that, as replicates declined the excessive (hyperbolic) increasing pattern of TBG fitted best in the four trials. Values of TBG increased smoothly with replicates declining up to 100, but a sharp increasing pattern was recorded with further replicate decline. Analysis of a particular entry size on different k constant (where applicable) resulted mostly in considerably different TBG values (Table 3). However, the gap was mitigated with the two trials of each set united (excepting the 31 entries’ case). For instance, in analysis of 13 entries TBG differed by 14 units (that is, 64 and 50 on k = 3 and 9, respectively) for ‘Evros’ and by 36 units for ‘Elassona’, but only by 5 units when the two trials were integrated. Values of TBG differed by 38 and 17 units when landrace ‘Lefkada’ was considered separately at 0.50 and 0.80 m, respectively, but just by 4 units when data were pooled across the two distance trials.

**Table 3:** Top to bottom gap for various number of entries (in parenthesis the k constant to construct the honeycomb design) allocated according to the design for each set of trials (the first comprising landraces Evros and Elassona and their united trial, while the second landrace Lefkada at interplant distance of 0.50 and 0.80 m and their united trial). The coefficient of linear correlation (*r*) between TBG and the corresponding entry size (n, number of plants) is also given

	1st set						2nd set					
Number of entries	Evros		Elassona		Pooled		Lefkada_50		Lefkada_80		Pooled	
	n	TBG	n	TBG	n	TBG	n	TBG	n	TBG	n	TBG
3	195	9	207	13	402	14	298	22	283	30	581	13
4	146	24	156	27	302	16	224	19	212	26	436	21
7 (2)^†^	83	26	89	39	172	27	128	61^†^	121	29	249	23
7 (4)	83	31	89	48^†^	172	23	128	23	121	33	249	20
9	65	27	69	71^‡^	134	30	99	51	94	61^‡^	193	32
12	49	77^‡^	52	78^‡^	101	45	75	56^‡^	71	78^‡^	146	40
13 (3)	45	64^‡^	48	44	93	51^‡^	69	69^‡^	65	55	134	47
13 (9)	45	50	48	80^‡^	93	46	69	46	65	64	134	39
16	37	77^‡^	39	53	76	52^‡^	56	54	53	74	109	43
19 (7)	31	74	33	84^‡^	64	55^‡^	47	86^‡^	45	65	92	53^‡^
19 (11)	31	75	33	108^‡^	64	56^‡^	47	47	45	61	92	43
21 (4)	28	62	30	122^‡^	58	55	43	72	40	110^‡^	83	76^‡^
21 (16)	28	96^‡^	30	98^‡^	58	61^‡^	43	110^‡^	40	93^‡^	83	80^‡^
25	23	64	25	150^‡^	48	71^‡^	36	130^‡^	34	107^‡^	70	86^‡^
27	22	105^‡^	23	85	45	64	33	86	31	85	64	77^‡^
28	21	109^‡^	22	78	43	75^‡^	32	114^‡^	30	104^‡^	62	80^‡^
31 (5)	19	161^‡^	20	109^‡^	39	94^‡^	29	103^‡^	27	149^‡^	56	63
31 (25)	19	149^‡^	20	105^‡^	39	69	29	96^‡^	27	121^‡^	56	80^‡^
*r*	−0.72***		−0.77***		−0.84***		−0.73***		−0.73***		−0.77***	

*** Significant at *p* < 0.001

^†^ k constant is given only for ‘ungrouped’ affecting the outcome of analysis

^‡^ Denotes at least one entry mean significantly differing from the grand mean at *p* < 0.05

**Fig. 4:**
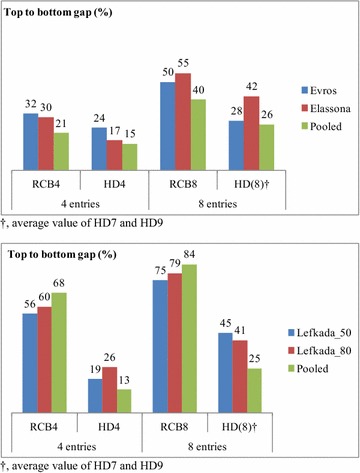
The upper graph demonstrates the top to bottom mean value gap as a percentage of the trial mean for yield per plant of landraces ‘Evros’, ‘Elassona’ and their unification concerning four and eight entries arranged according to either the randomized complete block (RCB) or the honeycomb design (HD), while at the lower graph the same applies for trials of landrace ‘Lefkada’ at 0.50 and 0.80 m and their unification. *Dagger* average value of HD7 and HD9

The honeycomb model compared with the classical RCB gave consistently lower TBG values (Fig. 4). Combination of the two trials resulted in lower TBG for both models in the first set but only for the honeycomb model in the second one. For the second set in particular, concerning four entries the average TBG value of 58 % increased to 68 % with pooled analysis for RCB, while for honeycomb analysis the average TBG value of 23 % reduced to 13 %. In terms of eight entries, the average TBG value of 77 % increased to 84 % with pooled analysis for RCB, while for honeycomb analysis the average TBG value of 43 % reduced to 25 %.

## Discussion

Grain legumes are fraught with uncertainty as they are sensitive to biotic and abiotic stresses increasing the spatial variability of yield [20]. Spatial heterogeneity due to stresses maximizes when plants are grown widely apart [10–14]. On the overall mean yields per plant and the respective CV values (Table 2), as well as the significant residuals of simulated entries (Table 3), it is obvious that huge spatial heterogeneity prevailed in the four trials of the three lentil landraces. Landrace ‘Lefkada’ was the most heterogeneous as further depicted in Fig. 3, and particularly at 0.80 m. Higher mean yield per plant by 30 % and lower CV by 14 % at the inter-plant distance of 0.50 compared to 0.80 m is an extra evidence that ‘Lefkada’ suffered the greatest spatial heterogeneity at 0.80 m. The three landraces of the study naturally evolving during farming in the past presumably comprised genetic variability; however, apparently the overwhelming majority of the variability recorded in this study was due to acquired plant-to-plant differences. The anomaly of acquired variability was a situation intended for specific breeding purposes since experimentation aimed to intensify virus infections seeking tolerant genotypes [13, 14], thus huge spatial heterogeneity was a reasonable consequence. Ultra-low densities favour insect landing [11], thus aphid-transmitted viruses lead to increased plant-to-plant variability [21].

The honeycomb pattern of experimentation is distinguishing for even and systematic entry allocation, thus one of the most appropriate tools to sample the spatial heterogeneity in field trials. It was found more effective compared to the classical models of random allocation (the RCB and the ‘nearest neighbour’ models), as well as that of the latice design [19]. Results of the current study were also supporting the honeycomb model against the RCB (Fig. 4). Nevertheless, it was hard to deal with such an extreme spatial heterogeneity exhibited by the lentil landraces, and the difficulties were pronounced with increased number of entries. In comparison with the previous study including a maize hybrid [19], honeycomb analysis resulted in much higher TBG values (e.g. 71 vs 7.5 % for nine entries) not fully attributable to the intra-landrace genetic variability. In addition, significant residuals appeared even in quite few entries, i.e. seven entries in ‘Elassona’ and ‘Lefkada’ at 0.50 m or nine entries in ‘Elassona’ and ‘Lefkada’ at 0.80 m or 12 entries in all the four trials. Moreover, under the atypical circumstances of this study k constant affected substantially the outcome of honeycomb analysis, contrary to the previous work where the k did not play any essential role [19].

Increasing the level of entries resulted in increased TBGs and significant residuals (Table 3). Larger area of the moving circular complete block to involve more entries reasonably inflates the mean differences because of greater intra-block variation. For instance, the interior circle in Fig. 1b (of both HD7 and HD19), that corresponds to the moving circular complete block involving seven entries, falls within a seemingly homogeneous area; however, the middle circle comprising 19 entries is obviously more heterogeneous, while even more heterogeneous is the exterior one including 31 entries. The size of the moving circular complete block was also the reason for the reduced success in ‘Lefkada’ at 0.80 compared with 0.50 m. At interplant distance of 0.50 m the area of the moving circular complete block of 7, 19 and 31 entries is 1.6, 4.3 and 7.1 m^2^, respectively; the respective areas at 0.80 m are 3.8, 10.5 and 17.2 m^2^ [18].

The number of entries is determined by the genotype and treatment, and limiting them is not always realistic. From the honeycomb experimentation perspective, considering unification of the two trials looks as though the number of replicates is crucial to remedy the spatial heterogeneity. Despite the extra experimental area that might reflect additional spatial heterogeneity, duplication of replicates inflated considerably the TBG values (Table 3). Figure 3 reveals that a number of replicates approaching 100 is the optimum threshold to smooth the extreme spatial heterogeneity prevailed in the four trials. Pooled data are further indicative (Table 3). From the point of statistics, spatial heterogeneity was counteracted up to 12 and 16 entries for the first and the second set of trials, respectively. The corresponding number of replicates was 101 for the former and 109 for the latter, while significant residuals appeared with replicate decline.

In the first set of trials, unification to increase replication improved effectiveness of either RCB or HD models for both four and eight entries; nevertheless, this was true only for HD in the second set (Fig. 4). In RCB trials, increasing the area of a block (usually of oblong square shape) very likely increases intrablock spatial heterogeneity. The statistical validity of the RCB and accurate estimates depend primarily on the assumption that treatments are evaluated with respect to similar environmental and operational conditions within a block [3–5, 22, 23]. Additionally, randomization is a crude technique given the complex patterns of spatial variability that exist, and there is no way to lay out blocks that will successfully account for spatial yield variability [19, 24]. On the other side, circular shape and standardized block type of the honeycomb model are two features that ensure the less possible extra heterogeneity with expanding the area of the block. The systematic entry arrangement vindicates the hypothesis that replication is in itself an attempt to account for the existence of spatial heterogeneity. Therefore, increased replicates per entry improve precision of evaluation and promote objectiveness of comparison, in agreement with the previous finding in maize and wheat trials [19]. Moreover, increased number of replicates bridged the gap between the outcome of different k constants (see Table 3 for 7, 13, 19, and 21 entries of pooled data vs component trials). Consequently, in the honeycomb breeding procedure when many entries are included the remedy against the heterogeneity of the higher block is the increase of the replicates per entry despite the concomitant greater overall spatial heterogeneity.

Inability to combat spatial variation could cause biased estimates of heritability [7, 25, 26], decreased response to selection and reduced precision of testing statistics [5]. In general, breeders are reluctant to use elaborating statistical designs particularly for characteristics other than yield [5], and prefer classical field-plot designs sustaining lower breeding efficiency [2, 7]. The necessity of elaborating statistical designs is imperative when huge spatial heterogeneity is avoidable and the honeycomb field layout seems appropriate to tackle such an adversity. Instead of the randomization in the classical models that bring about restrictions concerning the block size, the honeycomb model deals with the spatial heterogeneity in an exceptionally systematic way to cope with it effectively. Thanks to this attribute, breeders do not need to be concerned with the extra spatial heterogeneity that might induce the extra surface needed to expand the size of the block when many entries are considered. Instead, they could improve accuracy of comparisons with increasing the number of replicates per entry. The honeycomb breeding procedure in ‘Evros’ succeeded in potentially tolerant single-plant sister lines [13] and improved the sanitary status of seed stock in terms of seed-borne viruses [14], particularly concerning the most destructive *Pea seed*-*borne mosaic virus* (PSbMV); such an accomplishment has been realized in ‘Elassona’ and ‘Lefkada’ as well (data under consideration).

## Conclusions

Extremely high spatial heterogeneity occurred in the three lentil landraces mainly induced by virus infections. Under such adverse conditions the option of the elaborating honeycomb breeding model might be imperative, instead of the commonly used RCB, thanks to the systematic way of entry allocation. Despite the extra field surface, increasing the number of replicates per entry improved accuracy of comparisons. A number of 100 plants per progeny line was found optimum to mitigate the biased estimates and enhance breeding efficiency.

## Methods

Data from two sets of field trials (Table 1) were used pertaining to grain yield of individual plants of three lentil (*Lens culinaris* Medik.) landraces, named ‘Evros’, ‘Elassona’ and ‘Lefkada’. These trials are part of an ongoing project concerning breeding for tolerance to virus diseases, and extreme spatial variability due to severe infections [13, 14] rendered them appropriate from the perspective of this research effort. Spatial heterogeneity was approached by the TBG among the means of a number of simulated entries dispersed across the whole experimental area. The TBG was computed as a percentage of the overall trial mean. For example, the entry mean range of 70–125 % in relevance to the overall mean corresponds to 55 % TBG.

Two adjacent and similar trials were established according to the zig-zag arrangement of the honeycomb pattern, one for landrace ‘Evros’ and the other for landrace ‘Elassona’, with interplant distance of 1 m. Each trial comprised 34 rows of 32 single-plant hills, i.e., 1088 hills in total, corresponding to 30 m width (i.e. 34 × 1 × 0.87 m) and 32 m length, thus a total area of 960 m^2^.

The landrace ‘Lefkada’ was grown in two adjacent trials with interplant distance being 0.50 m in the first and 0.80 m in the second (i.e. ‘Lefkada_50’ and ‘Lefkada_80’). Each trial included 25 rows and 40 single-plant hills per row, approximating thus an experimental area of 220 and 576 m^2^. For this landrace in particular, the uniformity map on single-plant yields was constructed (Fig. 1). A considerable number of plants failed to give any seed because of intensified virus infection; thus to ensure representative values just for the construction of the uniformity map, the yield of each hill was corrected to the average yield of the respective moving circle of size 19 (i.e. the yield of code 7 was replaced by the average yield of the 19 plants included within the exterior circle). Thereafter the procedure of Petersen [27] described in Tokatlidis [19] was applied, smoothed in two directions. The trial was divided into units of four (2 × 2) plants and their average corrected yield was recorded: it comprised 20 rows of 13 means on each row, i.e. 260 yield PLs. The running median of three PLs was used whereby the yield of a PL was replaced by the median of the three adjacent PLs on the same row or the same column. Finally, areas of equal yield were delineated.

Each trial was divided subsequently into 4, 8, 16, and 32 equal PLs, defined as PL4, PL8, PL16, and PL32, respectively. As depicted in Fig. 1a that was accomplished via the three vertical continuous lines for PL4, plus the horizontal continuous line for PL8; further trial split through the two horizontal discontinuous lines resulted in PL16 and the extra four vertical discontinuous lines led to PL32. The PL16 was also considered as four horizontal randomized complete blocks of four entries (RCB4), while the PL32 as four horizontal randomized complete blocks of eight entries (RCB8). For RCB analysis, the TBG was computed in two conditions; in case (a) of the best randomization resulting in the lowest TBG and (b) of the worst randomization (on the premise that all the highest yielding PLs within blocks belonged to the same entry, all the PLs that gave the second highest yield belonged to the same entry, etc.).

On the honeycomb procedure, data were analyzed for possible configurations with regard to the 3–31 entries (HD3-31) constructed on the alternative k values (k is used so as to define the starting codes of each row) [18]. Those HDs including 4, 9, 12, 16, 25, 27, and 28 entries (that is, HD4, HD9, …, HD28) are classified “grouped” (the set of entries is split into more than one row), while HDs for 3, 7, 13, 19, 21, and 31 entries are “ungrouped” (entire set of entries is set on each row). In any “grouped” design potential k constant did not affect the outcome of honeycomb analysis (e.g. entry mean and standard deviation) as it was found in Tokatlidis [19] and this study as well. Therefore, different k constant was considered only for the “ungrouped” designs, excepted HD3 for which a single k applies (Table 3).

Data from the two trials of each set were united as if they were obtained from a single trial, i.e. 68 rows of 32 plants each for the first set and 50 rows of 40 plants each for the second set. Pooled data were analyzed as above for the honeycomb method, and as eight horizontal randomized complete blocks of four (RCB4) and eight (RCB8) entries.

### Statistical analysis

A computer program tailored to honeycomb designs was used for analysis of means (ANOM) [28]. Single-plant observations were subjected to the *t* test to appraise the significance of residual of each entry mean from the grand mean, i.e. $t = (\bar{x} - \bar{x}_{1} )/\sqrt {s^{2} /n}_{1}$ [29] where $\bar{x}$, and *s*, are mean and standard deviation of the overall population, while $\bar{x}_{1}$ is the entry mean and *n*_1_ its size (number of single-plant replicates). To approach the across-individuals phenotypic heterogeneity, CV on the single-plant basis was measured, e.g. for the overall population $CV = s/\bar{x}$. The relationship between the entry size and the magnitude of TBG was searched via the *Pearson* simple correlation and the data were adjusted to best fit excessive (hyperbolic) pattern (Fig. 3).

## Abbreviations

CV: coefficient of variation
HD: honeycomb design
PL: plot
RCB: randomized complete block
TBG: top to bottom gap
